# Soil chemical properties affect the reaction of forest soil bacteria to drought and rewetting stress

**DOI:** 10.1007/s13213-014-1002-0

**Published:** 2014-11-25

**Authors:** Marcin Chodak, Marcin Gołębiewski, Justyna Morawska-Płoskonka, Katarzyna Kuduk, Maria Niklińska

**Affiliations:** 1 Department of Management and Protection of Environment, AGH University of Science and Technology, al. Mickiewicza 30, 30-059 Kraków, Poland; 2 Department of Biotechnology, Nicolaus Copernicus University, ul. Gagarina 9, 87-100 Toruń, Poland; 3 Institute of Environmental Sciences, Jagiellonian University, Gronostajowa 7, 30-387 Kraków, Poland

**Keywords:** Drought and rewetting stress, Forest soils, Bacterial phyla, Soil chemical properties, Pyrosequencing

## Abstract

Reaction of soil bacteria to drought and rewetting stress may depend on soil chemical properties. The objectives of this study were to test the reaction of different bacterial phyla to drought and rewetting stress and to assess the influence of different soil chemical properties on the reaction of soil bacteria to this kind of stress. The soil samples were taken at ten forest sites and measured for pH and the contents of organic C (C_org_) and total N (N_t_), Zn, Cu, and Pb. The samples were kept without water addition at 20 – 30 °C for 8 weeks and subsequently rewetted to achieve moisture equal to 50 – 60 % of their maximum water-holding capacity. Prior to the drought period and 24 h after the rewetting, the structure of soil bacterial communities was determined using pyrosequencing of 16S rRNA genes. The drought and rewetting stress altered bacterial community structure. Gram-positive bacterial phyla, *Actinobacteria* and *Firmicutes*, increased in relative proportion after the stress, whereas the Gram-negative bacteria in most cases decreased. The largest decrease in relative abundance was for *Gammaproteobacteria* and *Bacteroidetes*. For several phyla the reaction to drought and rewetting stress depended on the chemical properties of soils. Soil pH was the most important soil property influencing the reaction of a number of soil bacterial groups (including all classes of *Proteobacteria*, *Bacteroidetes*, *Acidobacteria*, and others) to drought and rewetting stress. For several bacterial phyla the reaction to the stress depended also on the contents of N_t_ and C_org_ in soil. The effect of heavy metal pollution was also noticeable, although weaker compared to other chemical soil properties. We conclude that soil chemical properties should be considered when assessing the effect of stressing factors on soil bacterial communities.

**Electronic supplementary material:**

The online version of this article (doi:10.1007/s13213-014-1002-0) contains supplementary material, which is available to authorized users.

## Introduction

Soil bacteria constitute an important part of the soil microbial community and play a vital role in the functioning of forest ecosystems (Bauhus and Khanna 1999; Nannipieri et al. 2003). In forest soils, bacteria occur in largest abundance in the uppermost layers – the organic (O) and the accumulation (A) horizon (Raubuch and Beese 1995; Chodak 2002). Bacteria inhabiting the uppermost soil horizons are exposed to various external stressors, the long periods of drought followed by rapid rewetting being the most common ones (Schimel et al. 2007). Several climate models forecast more frequent and longer periods of drought in multiple forested regions of the world (IPCC 2007). Thus, drought and rewetting stress will likely become a more important perturbation to forest biogeochemical cycling in many regions (Maracchi et al. 2005).

Drought affects soil bacteria through osmotic stress and resource competition. Rewetting after a period of drought also causes stress for soil bacteria as they must rapidly dispose their osmolytes in order to counteract rapid flow of water into their cells (Schimel et al. 2007). Long periods of drought may lead to changes in the soil bacterial community structure (Schimel et al. 1999, 2007; Hueso et al. 2012) resulting in altered patterns of C and N cycling in the ecosystem. This is because some specific soil microbial processes are carried out by specialized bacteria that may be vulnerable to drought and rewetting stress (Evans and Burke 2012). For instance, Göransson et al. (2013) found lower rates of respiratory response to rewetting in drought-exposed forest soils compared with the soils that did not experience drought periods. Pesaro et al. (2004) reported reduced degradation and mineralization of organic pesticides in dried and rewetted soils compared to the degradation and mineralization in undisturbed control.

Different groups of soil bacteria may have different vulnerability to drought and rewetting stress (Uhlirova et al. 2005; Schimel et al. 2007) depending on their copiotrophic or oligotrophic character (Fierer et al. 2007) or their desiccation-related life-strategies (Barnard et al. 2013). However, studies on the reaction of different bacterial groups to the drought and rewetting stress usually neglected the importance of soil chemical properties for this process. It has been shown that taxonomic structure and diversity of soil bacterial communities are determined by soil properties such as quantity and quality of soil organic matter, moisture conditions or soil acidity (Allison and Martiny 2008; Rousk et al. 2010; Nacke et al. 2011; Kuramae et al. 2012; Preem et al. 2012; Tripahti et al. 2012; Chodak et al. 2013). We hypothesized, therefore, that the reaction of soil bacteria to drought and rewetting stress may depend not only on their intrinsic copiotrophic or oligotrophic characteristics or desiccation-related life-strategy, but may depend also on soil properties. In particular, the presence of other stressors such as high acidity or large heavy metal concentration could affect the reaction of soil bacteria to drought and rewetting stress.

The objectives of this study were to test the reaction of different bacterial phyla to drought and rewetting stress and to investigate how soil properties influence the reaction of different bacterial phyla to drought and rewetting.

## Material and Methods

### Study sites

Soil samples were collected at ten sites in southern Poland. In order to avoid a pseudoreplication problem (Hurlbert 1984), the sampling sites were located in two regions and at large distances from each other. Five sites were located in the Krakowsko-Częstochowska Upland nearby the city of Olkusz and another five in the Silesian Lowland between the cities of Legnica and Głogów. Within each region the five samples represented a heavy metal pollution gradient ranging from clean soils to heavily polluted ones.

The Krakowsko-Częstochowska Upland is made of Upper Jurassic rocks covered by Quaternary sands and loess. The annual precipitation averages 600 mm to 700 mm and the mean annual temperature is 8 °C. The Olkusz area is the major zinc and lead industry region of Poland; the country’s largest zinc smelter was built there in 1967. In the late 1980s, yearly dustfall in the vicinity of the smelter was ca. 118 tons km^−2^. Long-term industrial activity brought about severe pollution of forest soils with concentrations of Zn and Pb locally exceeding 4,600 mg kg^−1^ and 1,650 mg kg^−1^, respectively (Niklińska et al. 2005).

The Silesian Lowland is a large plain located in southwestern Poland. The sampling area was situated in its northwestern part. The climate of the area is temperate with mean annual temperature of 8.9 °C and annual precipitation averaging 500 – 550 mm. Since the late 1960s, the region has become a major copper production center in Poland. At present there are four copper ore mines and two copper smelters in the area. The industrial activity caused pollution of forest soils in the area with Cu and to some extent with Pb. The concentrations of these elements in organic horizons of forest soils reach nearly 1,200 mg kg^−1^ Cu and 515 mg kg^−1^ Pb (Niklińska et al. 2006).

### Soil sampling

The samples of organic (O) and A horizons were taken in June 2009 at ten sampling sites (area of each site ca. 1 ha). All the sampling sites were covered by Scots pine (*Pinus sylvestris*) forest stands (forest type: *Vaccinio Myrtilli* – *Pinetum*). The soils at the sampling sites were classified as Podzols developed from sands (clay content <5 %). The humus forms ranged from moder to mor (supplementary material Table S1). At each site the samples of O and A horizon were taken at five locations lying at the corners and in the middle of the sampling site. The samples from the five locations were sieved (10 mm and 2 mm mesh for O and A horizons, respectively) and transported to the laboratory. In the laboratory the samples were divided into two parts. One part was air-dried and used for physical, physico-chemical, and chemical analyses, and the other was stored in the dark, at 4 °C and used for microbial analyses.

### Chemical and physical analyses

The samples were analyzed for organic C (C_org_) and total N (N_t_) content by dry combustion using a CHNS analyzer (Vario EL III, Elementar Analysensysteme GmbH). The total concentrations of Zn, Pb, and Cu in the samples were measured after wet digestion in concentrated HNO_3_ with a gradual temperature increase from 50 °C to 150 °C using flame atomic absorption spectrometry with a graphite furnace technique (Perkin Elmer, AAnalyst 800). The pH was measured in 1 M KCl at a 1:2.5 ratio (sample:liquid, w:v) using a digital pH-meter (Nester Instr.). The texture of A horizon samples was determined using a hydrometer method (Sheldrick and Wang 1993). Maximum water-holding capacity (WHC) was determined gravimetrically according to Schlichting and Blume (1966).

### Drought and rewetting stress experiment

The soil samples from each site were pooled (on dry weigh basis) to form two mixed samples representative for O and A horizons at this site and used in a drought and rewetting stress experiment.

The mixed samples (ca. 100 g d.w.) were placed in plastic cups without a lid (vol. 125 ml) and supplemented with deionized water to achieve a moisture level equal to 50 – 60 % of their maximum WHC. The moistened samples were stored for a week in the dark at 22 °C to enable acclimation after sampling and pretreatment (Nielsen and Winding 2002). After this time, the subsamples were taken to measure initial taxonomic structure and diversity of soil bacterial communities. Subsequently, the samples were placed under drought conditions (temperature 20 °C/30 °C in 12-h intervals, no water addition) for 8 weeks. After this time, the samples were rewetted to obtain a moisture level equal to 50 – 60 % of their maximum WHC. The subsamples for measurement of soil bacterial community structure and diversity were taken 24 h after the rewetting.

### Microbial biomass estimation

Microbial biomass (C_mic_) was assessed with substrate-induced respiration (SIR) method (Anderson and Domsch 1978). Briefly, the fresh soil samples (5 g d.w. for O horizon and 50 g d.w. for A horizon) were adjusted to 50 ± 5 % of their maximum water-holding capacity, amended with 0.02 g glucose monohydrate g ^−1^ soil and incubated at 22 °C in gas-tight jars for 4 h for C_mic_. The jars contained small vessels with 5 ml 0.2 M NaOH to trap the evolved CO_2_. After opening the jars, 2 ml BaCl_2_ was added to the NaOH, and the excess hydroxide was titrated with 0.1 M HCl in the presence of phenolphthalein as indicator. C_mic_ was calculated from the SIR rate according to the equation: C_mic_ (mg g^−1^) =40.04 y +0.37, where y is given in ml CO_2_ h^−1^ g^−1^.

### Extraction of DNA from soils, PCR amplification, and pyrosequencing

The DNA was extracted from approximately 0.3 g of soil using a modified protocol of Yeates et al. (1997) based on bead beating cell lysis and SDS treatment, followed by polyethylene glycol (PEG)/sodium chloride and phenol:chloroform:isoamyl alcohol (25:24:1, v/v/v) nucleic acid extraction. The DNA extracted was quantified and checked for quality using a NanoDrop 1000 spectrophotometer (Nanodrop Technologies, Wilmington, DE, USA) and amplified using a polymerase chain reaction (PCR). In each PCR reaction 100 ng of the extracted DNA was used. For the PCR amplification of 16S rRNA genes of soil bacteria we used the forward amplicon fusion primer composed of the GS FLX Titanium B Primer sequence and the 27 F primer sequence (Liu et al. 2007), and the reverse amplicon fusion primer composed of the GS FLX Titanium amplicon A Primer, 8-bp-long Multiplex Identifier sequence (Hamady et al. 2008) and the 338R primer sequence (Liu et al. 2007). The PCR amplification was carried out in the Mastercycler (Eppendorf) in 20-μl reaction mixtures containing: 2 U of *Taq* Polymerase (Fermentas), 2 μl of 10 × PCR buffer with (NH_4_)_2_SO_4_ (Fermentas), 1.5 μM of each forward and reverse primer, 1.5 mM of MgCl_2_, 0.2 mM of each dNTP, 1.6 μl of bovine serum albumin (Fermentas), 10.1 μl of H_2_O, and 1 μl of diluted DNA. The PCR cycling scheme was the following: 95 °C for 3.5 min, followed by 34 cycles at 94 °C (30 s), 55 °C (1 min), 72 °C (1.5 min), and a final extension step at 72 °C for 10 min. Each sample was amplified in a separate reaction mixture containing a uniquely barcoded right primer, containing the MID sequence used in the next steps of analysis to assign sequences to particular samples. The DNA amplicons were mixed into approximately equimolar quantities based on the estimation of PCR product concentration trough agarose gel electrophoresis and purified using the MinElute PCR Purification Kit (QIAGEN). Purified pools were then sequenced as part of a single 454 FLX run according to the 454 Amplicon Sequencing protocols provided by the manufacturer (Roche 454) at the Institute of Biochemistry and Biophysics, Polish Academy of Sciences in Warsaw.

### Bioinformatic analyses, phylogenetic assignment, and clustering of 16S rRNA gene fragments

Raw reads were extracted from the .sff files with sff_extract with adapters clipping (−c) Blanca and Chevreux (2010). The downstream analyses were performed with the MOTHUR package v. 1.20.3 (Schloss et al. 2009). First, the reads were quality trimmed and reverse complemented, then the high quality set was dereplicated and aligned to the SILVA reference alignment. The aligned sequences were screened for those that did not cover the desired region of the alignment (start before column 2000, end on column 6326), and then the gap-only columns and columns containing at least one terminal gap symbol were filtered out of the alignment. The chimeras were identified using the Uchime algorithm (Edgar et al. 2011) implemented in MOTHUR. The sequences more numerous than the one being checked were used as possible chimera parents (Quince et al. 2001). PCR and residual sequencing noise were removed by Single Linkage Preclustering as described in Huse et al. (2007).

For average neighbor clustering-based operational taxonomic units (OTU), construction of a distance matrix was calculated on the basis of the final alignment with the dist.seqs command of MOTHUR, and OTUs were constructed with the cluster command of MOTHUR.

For community composition assessment, the high quality, non-chimeric reads were classified with classify.seqs, using the SILVA taxonomy file from the MOTHUR website. The assignment method was Bayesian.

The diversity of the bacterial communities was assessed using the Chao1 index calculated for OTUs with evolutionary distance of 0.03 (or 97 % 16S rRNA gene sequence similarity).

### Statistical analyses

Heavy metal pollution was expressed as toxicity index (TI) according to the following equation: TI = Zn_i_/EC_Zn_ + Cu_i_/EC_Cu_ + Pb_i_/EC_Pb_ where Zn_*i*_, Cu_*i*_ and Pb_*i*_ are the concentrations of Zn, Cu, and Pb at the *i*-th site and EC_Zn_, EC_Cu_ , and EC_Pb_ are the concentrations of Zn, Cu, and Pb causing 50 % reduction in dehydrogenase activity (Welp 1999).

The C_mic_, Chao1 index and relative abundances of most abundant bacterial phyla prior to and after the drought and rewetting stress averaged over ten sampling sites were compared using paired sample t-test.

Canonical correspondence analysis (CCA) was used to evaluate the influence of drought and rewetting stress on the structure of soil bacterial communities and to assess the effects of soil properties on the reaction of particular bacterial phyla to this kind of stress. In this analysis, we used the C_org_ and N_t_ contents as variables representing nutrient availability for the microbes, pH as a variable representing soil acidity, and TI as variable representing soil pollution with metals. The drought and rewetting stress was included as a dummy variable with 0 representing the microbial communities prior to the stress and 1 representing the communities after the stress.

The statistical analyses were performed with Statgraphics Centurion XVI software (StatPoint, Herndon, VA, USA) and the PAST program Hammer Ø et al. (2001).

## Results

### Chemical properties of the studied soils

The studied soils were polluted with different loads of Cu, Zn, and Pb. In the O horizon, the highest contents of these metals were 1,353 mg kg^−1^, 4,792 mg kg^−1^, and 1,877 mg kg^−1^, respectively and the lowest 2 mg kg^−1^, 54 mg kg^−1^, and 177 mg kg^−1^, respectively (Table 1). In the A horizon heavy metal contents were approximately ten times lower and reached maximum values of 98.4 mg kg^−1^, 422.4 mg kg^−1^, and 180.8 mg kg^−1^. Copper was the main pollutant in the soils of the Legnica region whereas the soils of the Olkusz region were polluted mainly with Zn and Pb. The calculated TI values in the O horizon ranged from 2.1 to 46.1 and in the A horizon from 0.2 to 4.1. The studied soils were strongly acidic (pH =3.4 – 5.7) (Table 1). The contents of C_org_ and N_t_ were much higher in the O horizon than in the A horizon but the C_org_-to-N_t_ ratios were similar in both horizons and varied from 19.6 to 45.4.

**Table 1:** Chemical properties of soils

Horizon	Site	Cu	Zn	Pb	Toxicity index	pH	N_t_	C_org_	C_org_-to-N_t_
		mg kg^−1^					mg g^−1^		
O	OLK1	52	4,792	1,877	46.1	5.6	6.48	160.3	24.7
	OLK2	28	1,946	1,319	19.7	5.0	10.87	246.0	22.6
	OLK3	10	818	747	8.6	4.7	7.11	185.3	26.1
	OLK4	2	294	404	3.2	4.4	12.96	337.6	26.0
	OLK5	3	193	198	2.1	4.8	8.14	214.2	26.3
	LEG1	1,353	116	778	40.9	3.8	14.15	393.8	27.8
	LEG2	999	77	506	30.0	4.1	9.94	264.2	26.6
	LEG3	410	54	241	12.5	3.8	12.56	361.4	28.8
	LEG4	217	74	177	7.1	3.8	11.66	392.5	33.7
	LEG5	85	56	191	3.2	3.4	11.90	428.1	36.0
A	OLK1	6.2	422.4	155.3	4.1	5.7	0.42	16.73	40.1
	OLK2	1.2	100.0	180.8	1.2	5.2	0.40	17.11	43.0
	OLK3	1.3	86.7	78.7	0.9	5.2	0.44	16.05	36.3
	OLK4	0.5	29.6	108.7	0.4	4.6	1.66	53.31	32.2
	OLK5	0.0	45.6	66.9	0.5	5.0	0.67	24.83	37.1
	LEG1	98.4	22.2	51.1	3.1	3.9	0.43	12.53	28.9
	LEG2	33.5	15.1	18.6	1.1	4.6	1.84	36.06	19.6
	LEG3	12.5	11.7	21.6	0.5	4.3	0.73	19.69	27.1
	LEG4	29.7	13.6	44.7	1.0	4.1	1.43	35.87	25.1
	LEG5	2.5	10.4	26.7	0.2	4.2	0.60	27.21	45.4

### The structure and diversity of soil bacterial communities prior to and after the drought and rewetting stress

The C_mic_ values prior to stress varied from 0.64 mg g^−1^ to 2.85 mg g^−1^ in the O horizon, and from 0.11 mg g^−1^ to 0.24 mg g^−1^ in the A horizon (Table 2). After the stress, C_mic_ decreased significantly in both horizons (p <0.05) and varied from 0.61 mg g^−1^ to 2.75 mg g^−1^ and from 0.08 mg g^−1^ to 0.22 mg g^−1^ in the O and A horizons, respectively (Table 2).

**Table 2:** Microbial biomass (C_mic_) and Chao1 diversity index in the O and A horizons at different sites prior to and after the drought and rewetting stress. Asterisks denote significant difference between the values prior to and after the stress within the horizon (p <0.05, pairwise t-test)

Prior to drought and rewetting stress (T1)			After drought and rewetting stress (T2)	
O Horizon				
Site	C_mic_ (mg g^−1^)	Chao1	C_mic_ (mg g^−1^)	Chao1
OLK1	0.64	2,679	0.61	2,266
OLK2	1.29	3,110	1.17	1,682
OLK3	1.06	1,953	0.97	1,032
OLK4	2.85	2,249	2.75	2,097
OLK5	1.33	2,499	1.26	2,670
LEG1	1.75	795	1.59	639
LEG2	1.09	628	0.94	411
LEG3	1.29	607	1.15	473
LEG4	1.91	860	1.65	563
LEG5	1.38	912	1.29	n.d.
Mean	1.46^*^	1,629^*^	1.34^*^	1,315^*^
A Horizon				
OLK1	0.23	2,337	0.20	2,261
OLK2	0.22	2,974	0.18	1,061
OLK3	0.18	2,174	0.17	1,739
OLK4	0.21	1,438	0.18	1,827
OLK5	0.21	3,104	0.21	2,112
LEG1	0.24	778	0.22	589
LEG2	0.11	633	0.08	705
LEG3	0.14	581	0.13	575
LEG4	0.22	1,177	0.21	790
LEG5	0.13	648	0.13	473
Mean	0.19^*^	1,584	0.17^*^	1,213

n.d. – not determined

Prior to the stress, the values of Chao1 index varied between 607 and 3,110 and were much higher in the soils from the Olkusz region than in the soils from the Legnica region. After the stress, the Chao1 index values declined, although only in the O horizon was the decrease statistically significant (Table 2).

**Fig. 1:**
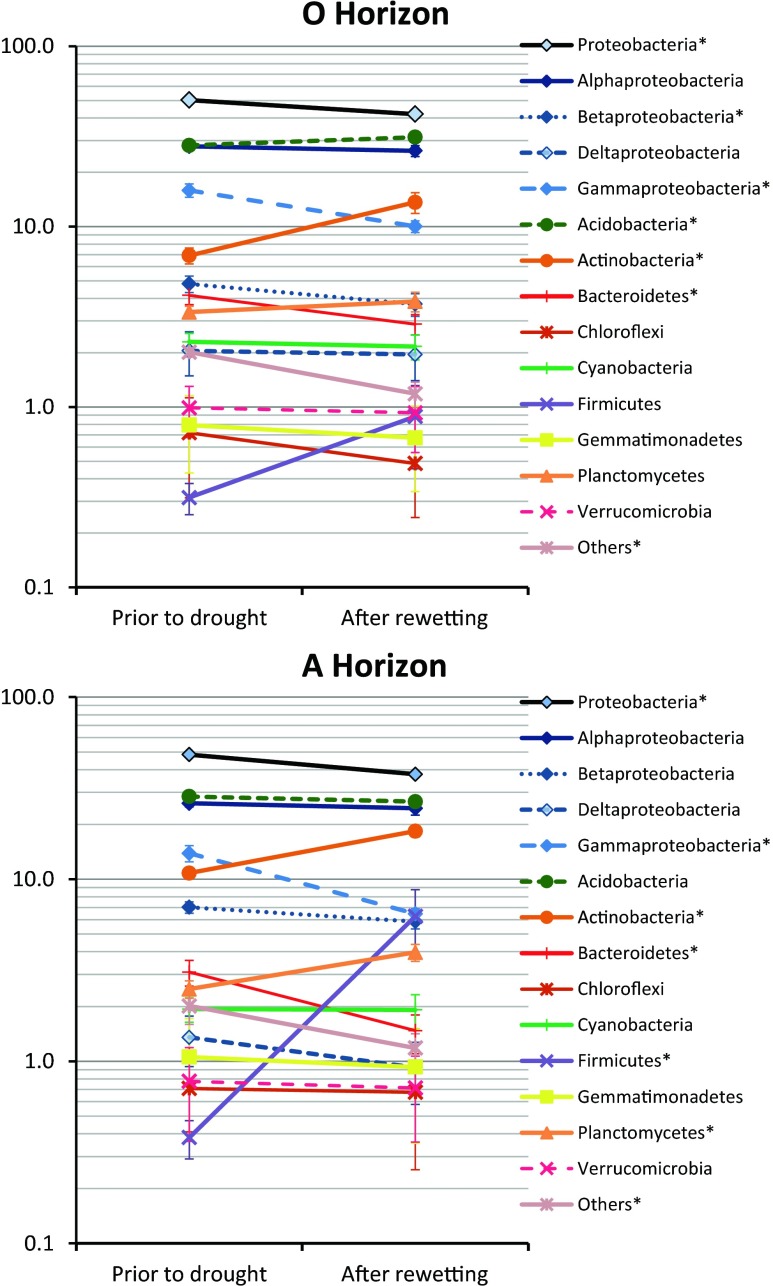
The relative abundances of most abundant bacterial phyla in the O and A horizons prior to and after the drought and rewetting stress. Data averaged over ten sampling sites, bars indicate standard errors. Asterisks denote bacterial phyla with different relative abundance prior to and after the stress (p < 0.05, paired samples t-test)

In both horizons prior to the stress, the dominating bacterial phylum was *Proteobacteria*, which constituted up to 57.5 % of the total OTUs detected. Among *Proteobacteria* the most abundant was the *Alphaproteobacteria* class, followed by *Gammaproteobacteria* and *Betaproteobacteria* (Fig. 1, supplementary material Table S2 and S3). Large shares were found also for *Acidobacteria* (up to 34.6 % of the total OTUs detected), *Actinobacteria* (up to 15.2 % of the total OTUs detected), *Bacteroidetes* (up to 7.0 % of the total OTUs detected) and *Planctomycetes* (up to 4.2 % of the total OTU’s detected). The other bacterial phyla were less abundant (Fig. 1, Tables S2 and S3).

**Fig. 2:**
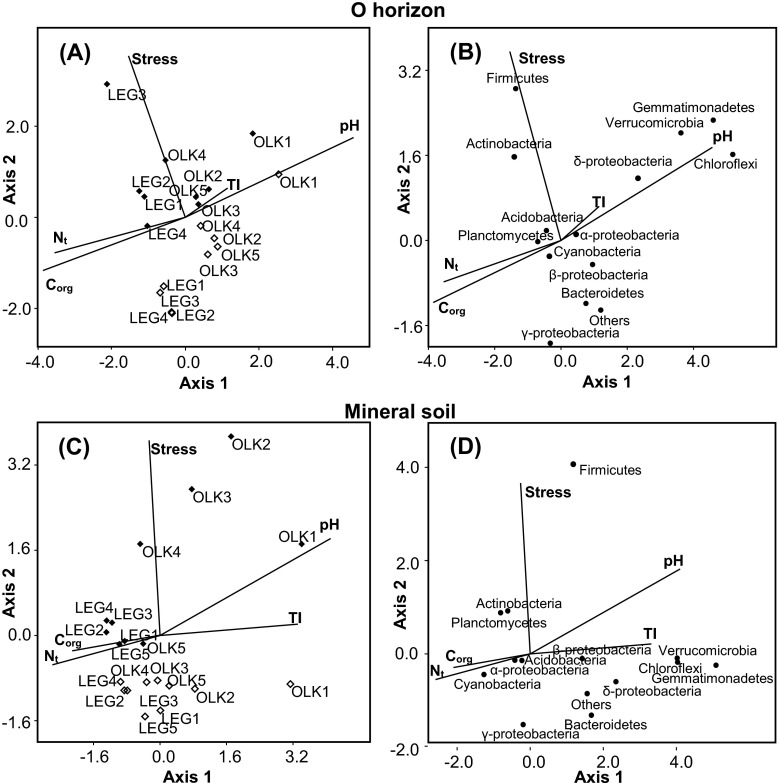
The canonical correspondence analysis (CCA) loading plot of the bacterial structure composition in relation to soil properties and the drought and rewetting stress in the O horizon (**a**) and mineral soil (**c**) and the ordination plots of the relationships between bacterial phyla and soil properties in the O horizon (**b**) and mineral soil (**d**) for forest soils sampled in two regions (OLK – Olkusz region, LEG – Legnica region). Soil properties include toxicity index (TI), soil pH in KCl (pH), contents of organic C (C_org_), total N (N_t_). Drought and rewetting stress included as “Stress” variable. In the ordination plots, empty diamonds denote the sites prior to and filled diamonds the sites after the stress

The drought and rewetting stress changed the structures of soil bacterial communities in both soil horizons as indicated by altered average abundances of some bacterial groups (Fig. 1) and by distinct shifts of site points along the stress vectors in the CCA biplots (Fig. 2).

After the drought and rewetting stress, the average share of *Proteobacteria* phylum decreased significantly in both soil horizons (Fig. 1). Different classes of *Proteobacteria* were affected differently by the drought and rewetting stress with *Gammaproteobacteria* showing the largest decrease in both soil horizons studied (Figs. 1 and 2). In the O horizon, the share of *Gammaproteobacteria* decreased from 15.9 % to 10.0 %. In the A horizon, the decrease was even larger (from 13.9 % to 6.5 %) (Fig. 1).

Negative effect of the drought and rewetting stress was observed also for *Bacteroidetes* and bacteria classified as others, representing less abundant taxons (Fig. 2). The shares of *Chloroflexi*, *Gemmatimonadetes* and *Verrucomicrobia* were negatively affected by drought and rewetting stress, although abundances of these bacterial phyla depended mainly on soil pH (Fig. 2).

In both studied soil horizons after the drought and rewetting stress distinctly increased the shares of *Firmicutes* and *Actinobacteria* (Figs. 1 and 2). Positive effect of the drought and rewetting stress on the relative abundance was observed also for *Acidobacteria* in the O horizon and for *Planctomycetes* in the mineral soil (Fig. 2).

### Relationships between soil chemical properties and the reaction of bacterial phyla to drought and rewetting stress

The first two CCA axes calculated for the O horizon explained 63.1 % (p =0.001) and 19.6 % (p =0.02) of the variance, respectively. For the A horizon, the first CCA axis explained 53.7 % (p =0.04) and the second axis 37.3 % (p =0.01) of the variance. In the O horizon the first CCA axis was strongly related to pH (0.91), C_org_ (−0.77), and N_t_ (−0.71). However, there was also a distinct relationship with the drought and rewetting stress (−0.31). The drought and rewetting stress was strongly related (0.71) to the second CCA axis. However, pronounced relationships were also between the CCA axis 2 and soil pH (0.35), C_org_ (−0.23) content, N_t_ (−0.16) content, and TI (0.13). In the mineral soil, the drought and rewetting stress was strongly related to the second CCA axis (0.73). Among the soil properties, only pH was distinctly related to this axis (0.36), whereas the relationships of contents of C_org_ (−0.05), N_t_ (−0.11), and TI (0.04) were weak.

## Discussion

### The structure of soil bacterial community prior to the drought and rewetting stress

Bacterial communities in both horizons of the studied forest soils were dominated by *Proteobacteria* followed by *Acidobacteria* and *Actinobacteria*. In several studies, by applying different molecular methods, *Proteobacteria*, *Acidobacteria*, and *Actinobacteria* were reported to be the most abundant bacterial phyla (Janssen 2006). In our soils, among *Proteobacteria* the class *Alphaproteobacteria* was the most abundant one. A high proportion of *Alphaproteobacteria* was reported for various deciduous and coniferous forests (Nacke et al. 2011; Kuramae et al. 2012) and seems to be characteristic for forest soil bacterial communities.

The second most abundant phylum in our soils was *Acidobacteria*. A high proportion of *Acidobacteria* was found in boreal forest soils (Dimitriu and Grayston 2010), acid soils of temperate forests, as well as in grassland and pasture soils (Nacke et al. 2011). A large abundance of *Acidobacteria* in different soils indicates their importance for functioning of soils. However, little is known about physiology and metabolic functions of *Acidobacteria*. Fierer et al. (2007) described *Acidobacteria* as oligotrophs, favored in poor soils with lower carbon availability. The soils used in our study were acid, sandy textured, with low contents of nutrients and poor water holding capacity. Low fertility of our soils explains therefore the high share of *Acidobacteria*.

The other phyla such as *Bacteroidetes*, *Firmicutes*, *Planctomycetes*, or others were less abundant in our soils. The studied soils differed in terms of soil bacterial community structure and the main drivers of the observed differences have been described elsewhere (Chodak et al. 2013).

The general composition of bacterial communities did not differ much between the O and A horizons. There were only few interlayer differences in the shares of some taxa. The O horizons contained higher proportions of *Gammaproteobacteria* and *Planctomycetes* and a lower proportion of *Actinobacteria*. Analogous results have been reported by Dimitriu and Grayston (2010) who found similar bacterial composition in organic and mineral layers in boreal soils. The lack of distinct soil horizon effect on the structure of soil bacterial community may be attributed to the confounding effect of topography (affecting the patterns of nutrient and moisture contents in organic and mineral horizons) and downward flow of extracellular DNA (Dimitriu and Grayston 2010).

### The effect of drought and rewetting stress on diversity and structure of bacterial communities

Drought and rewetting stress caused a small or moderate decrease of microbial biomass in the studied soils. Drought periods are known to cause microbial biomass decline in forest soils (Schimel et al. 1999). However, drought and rewetting is the most frequently occurring environmental stress, and the most common soil bacteria have developed several survival mechanisms (Schimel et al. 2007; Barnard et al. 2013). This may explain the small decrease of microbial biomass in our studied soils. The decline of microbial biomass was accompanied by a small loss of bacterial diversity indicating that despite various survival mechanisms available to soil bacteria, some of them are vulnerable to this kind of stress and may become entirely extinct after prolonged drought.

Different bacterial phyla were differently affected by the drought and rewetting stress resulting in considerable change in the structure of soil bacterial communities. The obtained results confirmed that Gram-positive bacteria are more resistant to drought and rewetting stress than the Gram-negative bacteria (Schimel et al. 2007). Increased shares after the stress, were found for *Actinobacteria* and *Firmicutes. Actinobacteria* and *Firmicutes* are Gram-positive bacteria and many of them are known to form spores, which are resistant to desiccation and can survive under drought conditions (Singh et al. 2007; Zhang and Xu 2008). These two bacterial phyla could not be classified either as oligo- or copiotrophs by Fierer et al. (2007). However, Barnard et al. (2013) reported that desiccation stimulated ribosomal synthesis of *Actinobacteria,* and this may explain their higher relative abundance after the drought and rewetting stress in our study.

The increased shares after drought and rewetting stress were found also for *Acidobacteria*. However, this effect was evident only in the O horizon. In the A horizon, the reaction of *Acidobacteria* to the stress was variable and highly dependent on soil pH. In more acid soils (pH < ∼5) the relative abundance of *Acidobacteria* increased after the stress, whereas in the less acid ones it decreased. *Acidobacteria* are oligotrophs well adapted to acid soils and probably that is why at lower pH they were better able to survive the drought and rewetting stress compared to other bacterial phyla (Ward et al. 2009). At higher pH values, however, other phyla – in particular, *Firmicutes* and *Betaproteobacteria* – proved more resistant and increased their relative abundance after the drought and rewetting stress.

Gram-negative bacteria generally decreased in relative abundance after drought and rewetting stress. The only Gram-negative phylum that appeared resistant to drought and rewetting stress was *Planctomycetes,* which increased in relative abundance after the stress in the A horizon. *Planctomycetes* are slow growing, aerobic bacteria that have several unusual characteristics: they lack peptidoglycan in their cell walls, their cells are divided by inner membranes into several compartments, and their genomes tend to be large (Buckley et al. 2006). Our result suggests that they are more resistant to drought and rewetting stress than other Gram-negative bacteria.

The relative abundance of *Proteobacteria* and *Bacteroidetes* decreased after drought and rewetting stress. These two phyla represent Gram-negative bacteria characterized by high sensitivity to environmental disturbances and vulnerability to drought stress (Uhlirova et al. 2005; Schimel et al. 2007; Barnard et al. 2013). They are mostly considered r-strategists decreasing in numbers under unfavorable conditions and boosting when the conditions improve (Lu et al. 2006; Singh et al. 2007). Among *Proteobacteria*, the class of *Gammaproteobacteria* was most severely affected by drought and rewetting stress. *Gammaproteobacteria* play a major role in important biogeochemical processes being involved in decomposition of numerous C compounds including phenols, PAHs, alkaloids, and carbohydrates (Abd-El-Haleem et al. 2002; Padmanabhan et al. 2003; Cleveland et al. 2007). Cleveland et al. (2007) suggested that *Gammaproteobacteria* may be disproportionally important to the decomposition of labile C compounds in soils. High vulnerability of *Gammaproteobacteria* to drought and rewetting stress implies that prolonged drought periods followed by rapid rewetting may have a strong impact on C cycling in temperate forest soils.

In both studied soil horizons, the drought and rewetting stress decreased significantly the relative proportion of less abundant bacterial taxa referred to as others. Distinct decline of these rare bacterial species indicates their vulnerability to drought and rewetting stress. The importance of rare bacterial taxa for soil functioning is unknown (Gans et al. 2005; Hol et al. 2010). However, rare species can play essential roles in soil functioning when they perform specific functions. The importance of rare bacterial species has been highlighted by Hol et al. (2010), who reported that rare soil bacteria were involved in complex community relationships affecting plant productivity and interactions between plants and herbivores.

The method applied to study structure of soil bacterial communities was based on the analysis of bacterial DNA extracted from the soil; therefore, the obtained results may be biased by the presence of DNA from dead microorganisms (Wackernagel 2006). However, microbial DNA is relatively rapidly decomposed when released to soil, in particular at early stages of its decomposition (Wackernagel 2006). DNA may be protected in soils owing to binding to clay minerals (Nielsen et al. 2006). However, our soils contained only small amounts of clay particles. Therefore, we presume that the contribution of DNA from dead bacteria in our study was negligible, and the observed changes result from the stress-induced alteration of soil bacterial communities.

### The effect of soil properties on the reaction of particular bacterial phyla to drought and rewetting stress

The effect of drought and rewetting on soil microbial properties has been extensively studied (Schimel et al. 1999; Uhlirova et al. 2005; Hueso et al. 2012; Barnard et al. 2013). However, the role of soil chemical properties as factors that may modify the reaction of soil bacteria to this kind of stress received less attention. The CCA analysis applied in our study revealed that the reaction of several bacterial phyla to drought and rewetting stress depended on chemical soil properties. In particular, strong effect on the reaction of several bacterial groups to the applied stress was from soil pH. The effect of soil acidity was evident in both studied soil horizons. Soil pH has been described as a powerful control of soil microorganisms (Lauber et al. 2009; Rousk et al. 2010; Tripahti et al. 2012) affecting diversity and structure of soil microbial communities either directly (Rousk et al. 2010) or indirectly through changes in carbon and nutrient availability (Kemmitt et al. 2006). Our results suggest that the effect of soil pH on the structure of soil bacterial communities may be through altering the reaction of particular bacterial groups to commonly occurring environmental stressors.

Strong influence of soil pH on the reaction of some bacterial groups to drought and rewetting stress suggests that the effect of prolonged drought periods on microbial processes may differ depending on soil acidity. For instance, in the O horizon we found that the decline of *Gammaproteobacteria*, *Bacteroidetes,* and rare bacterial taxons (“others”) was larger at lower pH values. In consequence, stronger negative effects on the soil process driven by these bacteria should be expected in more acid soils compared with the less acid and neutral ones. In particular, decomposition of labile C compounds may be seriously affected in more acid soils due to the decrease of *Gammaproteobacteria* that are highly important for this process (Padmanabhan et al. 2003).

Differences in soil pH may lead to different reactions to drought and rewetting stress of the same bacterial groups. For instance, in the A horizon we have observed that in more acid soils the share of *Betaproteobacteria* decreased after the stress decreased, whereas in less acid ones it increased. For *Alphaproteobacteria* and *Acidobacteria*, the opposite was the case. Such a variable reaction of some bacterial groups may impede their ecological classification, and therefore, soil pH should always be considered when assessing the effects of stressing factors on different groups of soil bacteria.

The reaction of soil bacteria to drought and rewetting stress was related not only to soil pH, but also to the contents of C_org_ and N_t_. This suggests that the reaction to the drought and rewetting stress may depend on the content of nutrients (N_t_) and energy source (C_org_) in soil. However, the C_org_ and N_t_ contents were inversely related to soil pH. Therefore, we cannot rule out that the effect of C_org_ and N_t_ contents was at least partly due to their negative correlation with soil pH. It is known that low pH may result in retarded organic matter decomposition in soils (Kemmitt et al. 2006). In such a case, the influence of C_org_ and N_t_ contents for the reaction of soil bacteria to drought and rewetting stress would be another expression of the pH effect.

The heavy metal pollution also affected the reaction of soil bacteria to drought and rewetting stress. However, despite large concentrations of heavy metals in some of the studied soils, their effect was weaker compared to other soil properties. This demonstrates that even in areas highly polluted with heavy metals, natural soil properties such as pH or nutrient contents may be more important than the pollution in determining some properties of microbial communities. In line with our results, Azarbad et al. (2014) reported a minor effect of heavy metal pollution on several functional gene families related to i.a. stress responses in microbial communities from soils polluted with high levels of Pb, Zn, and Cd.

## Conclusions

Long periods of drought followed by rapid rewetting decreased microbial biomass, diversity of soil bacterial communities, and brought about changes in bacterial community structure. Different bacterial phyla differed in their vulnerability to this kind of stress. In general, Gram-positive bacterial phyla – *Actinobacteria* and *Firmicutes* – were more resistant to drought and rewetting stress than the Gram-negative bacteria. The copiotrophic, Gram-negative bacteria such as *Proteobacteria* and *Bacteroidetes* were more vulnerable and usually decreased in share after the drought and rewetting. However, their reaction depended on the soil properties – in particular on soil pH. Low soil pH reduced the ability of copiotrophic *Gammaproteobacteria* and *Bacteroidetes* as well as the rare bacterial species to withstand drought and rewetting stress. Since these bacteria (in particular *Gammaproteobacteria*) are responsible for important soil processes the drought and rewetting stress may have stronger effect on the functioning of more acid soils compared with the less acid and the neutral ones.

The reaction of several other groups of bacteria to drought and rewetting stress depended also on various soil properties such as the contents of N and organic C and the heavy metal pollution. Therefore, chemical properties of soils should always be regarded when assessing the effect of drought and rewetting on soil bacterial communities.

## Electronic supplementary material

Below is the link to the electronic supplementary material.

ESM 1 (DOCX 14 kb)

ESM 2 (DOCX 19 kb)

ESM 3 (DOCX 18 kb)

## References

[CR1] Abd-El-Haleem D, Moawad H, Zaki EA, Zaki S (2002). Molecular characterization of phenol-degrading bacteria isolated from different Egyptian ecosystems. Microb Ecol.

[CR2] Allison SD, Martiny JBH (2008). Resistance, resilience and redundancy in microbial communities. Proc Natl Acad Sci U S A.

[CR3] Anderson JPE, Domsch KH (1978). A physiological method for the quantitative measurement of microbial biomass in soils. Soil Biol Biochem.

[CR4] Azarbad H, Niklińska M, Laskowski R, van Straalen NM, van Gestel CAM, Zhou J, He Z, Wen C, Röling WFM (2014) Microbial community composition and functions are resilient to metal pollution along two forest soil gradients. FEMS Microbiology Ecology. *In press*10.1093/femsec/fiu003 25764529

[CR5] Barnard RL, Osborne CA, Firestone MK (2013) Responses of soil bacterial and fungal communities to extreme desiccation and rewetting. The ISME Journal, 1 – 13, doi:10.1038/ismej2013104 10.1038/ismej.2013.104 PMC3806258 23823489

[CR6] Bauhus J, Khanna PK, Rastin N, Bauhus J (1999). The significance of microbial biomass in forest soils. Going Underground - Ecological Studies in Forest Soils.

[CR7] Blanca J, Chevreux B (2010) bioinf.comav.upv.es/sff_crumbs/

[CR8] Buckley DH, Huangyutitham V, Nelson TA, Rumberger A, Thies JE (2006). Diversity of *Planctomycetes* in soil in relation to soil history and environmental heterogeneity. Appl Environ Microbiol.

[CR9] Chodak M (2002) Chemical and biological characteristics of organic layers under spruce and beech stands. Berichte des Forschungszentrums Waldökosysteme, Reihe A, Bd180

[CR10] Chodak M, Gołębiewski M, Morawska-Płoskonka J, Kuduk K, Niklińska M (2013). Diversity of microorganisms from forest soils differently polluted with heavy metals. Appl Soil Ecol.

[CR11] Cleveland CC, Nemergut DR, Schmidt SK, Townsend AR (2007). Increases in soil respiration following labile carbon additions linked to rapid shifts in soil microbial community composition. Biogeochemistry.

[CR12] Dimitriu PA, Grayston SJ (2010). Relationship between soil properties and patterns of bacterial β-diversity across reclaimed and natural boreal forest soils. Microb Ecol.

[CR13] Edgar RC, Haas BJ, Clemente JC, Quince C, Knight R (2011). UCHIME improves sensitivity and speed of chimera detection. Bioinformatics.

[CR14] Evans SE, Burke IC (2012). Carbon and nitrogen decoupling under an 11-year drought in the shortgrass steppe. Ecosystems.

[CR15] Fierer N, Bradford MA, Jackson RB (2007). Toward an ecological classification of soil bacteria. Ecology.

[CR16] Gans J, Wolinsky M, Dunbar J (2005). Computational improvements reveal great bacterial diversity and high metal toxicity in soil. Science.

[CR17] Göranson H, Godbold DL, Jones DL, Rousk J (2013) Bacterial growth and respiration responses upon rewetting dry forest soils: Impact of drought legacy. Soil Biol Biochem 57: 477–486

[CR18] Hamady M, Walker J, Harris JK, Gold NJ, Knight R (2008). Error-correcting barcoded primers for pyrosequencing houndreds of samples in multiplex. Nat Methods.

[CR19] Hammer Ø, Harper DAT, Ryan PD (2001) PAST: Paleontological Statistics Software Package for Education and Data Analysis. Palaeontologia Electronica 4: 9 pp

[CR20] Hol WHG, de Boer W, Termorshuizen AJ, Meyer KM, Schneider JH, van Dam NM, van Veen JA, van der Putten WH (2010). Reduction of rare soil microbes modifies plant-herbivore interactions. Ecol Lett.

[CR21] Hueso SC, García C, Hernández T (2012). Severe drought conditions modify the microbial community structure, size and activity in amended and unamended soils. Soil Biol Biochem.

[CR22] Hurlbert SH (1984). Pseudoreplication and the design of ecological field experiments. Ecol Monographs.

[CR23] Huse SM, Huber JA, Morrison HG, Sogin ML, Welch DM (2007). Accuracy and quality of massively parallel DNA pyrosequencing. Genome Biol.

[CR24] Solomon S, Qin D, Manning M, IPCC (2007). Climate change 2007: the physical science basis. Contribution of working group I to the fourth assessment report of the intergovernmental panel on climate change.

[CR25] Janssen PH (2006). Identifying the dominant soil bacterial taxa in libraries of 16S rRNA and 16S rRNA genes. Appl Environ Microbiol.

[CR26] Kemmitt SJ, Wright D, Goulding KWT, Jones DL (2006). pH regulation of carbon and nitrogen dynamics in two agricultural soils. Soil Biol Biochem.

[CR27] Kuramae EE, Yergeau E, Wong LC, Pijl AS, van Veen JA, Kowalchuk GA (2012). Soil characteristics more strongly influence soil bacterial communities than land-use type. FEMS Microbiol Ecol.

[CR28] Lauber CL, Hamady M, Knight R, Fierer N (2009). Pyrosequencing-based assessment of soil pH as a predictor of soil bacterial community structure at the continental scale. Appl Environ Microbiol.

[CR29] Liu Z, Lozupone C, Hamady M, Bushman FD, Knight R (2007). Short pyrosequencing reads suffice for accurate microbial community analysis. Nucleic Acids Res.

[CR30] Lu Y, Rosencrantz D, Liesack W, Conrad R (2006). Structure and activity of bacterial community inhabiting rice roots and the rhizosphere. Environ Microbiol.

[CR31] Maracchi G, Sirotenko O, Bindi M (2005). Impacts of present and future climate variability on agriculture and forestry in the temperate regions: Europe. Clim Change.

[CR32] Nacke H, Thürmer A, Wollherr A, Will C, Hodac L, Herold N, Schöning I, Schrumpf M, Daniel R (2011) Pyrosequencing-based assessment of bacterial community structure along different management types in German forest and grassland soils. PLoS ONE 6(2): e17000 doi:10.1371/journalpone0017000 10.1371/journal.pone.0017000 PMC3040199 21359220

[CR33] Nannipieri P, Ascher J, Ceccherini MT, Landi L, Pietramellara G, Renella G (2003). Microbial diversity and soil functions. Eur J Soil Sci.

[CR34] Nielsen MN, Winding A (2002). Microorganisms as indicators of soil health. NERI Tech Rep.

[CR35] Nielsen KM, Calamai L, Pietramellara G (2006) Stabilization of Extracellular DNA and Proteins by Transient Binding to Various Soil Components. In: Nucleic Acids and Proteins in Soil, Nannipieri P and Smalla C (Eds), Soil Biology Vol 8, Springer, pp 141 – 158

[CR36] Niklińska M, Chodak M, Laskowski R (2005). Characterization of the forest humus microbial community in a heavy metal polluted area. Soil Biol Biochem.

[CR37] Niklińska M, Chodak M, Laskowski R (2006). Pollution-induced community tolerance of microorganisms from forest soil organic layers polluted with Zn or Cu. Appl Soil Ecol.

[CR38] Padmanabhan P, Padmanabhan S, DeRito C, Gray A, Gannon D, Snape JR, Tsai CS, Park W, Jeon C, Madsen EL (2003). Respiration of ^13^C-labelled substrates added to soil in the field and subsequent 16S rRNA gene analysis of ^13^C-labelled soil DNA. Appl Environ Microbiol.

[CR39] Pesaro M, Nicollier G, Zeyer J, Widmer F (2004). Impact of soil drying-rewetting stress on microbial communities and activities and on degradation of two crop protection products. Appl Environ Microbial.

[CR40] Preem J-K, Truua J, Truu M, Mandera Ü, Oopkaupa K, Lohmus K, Helmisaari H-S, Uri V, Zobel M (2012). Bacterial community structure and its relationship to soil physico-chemical characteristics in alder stands with different management histories. Ecol Eng.

[CR41] Quince C, Lanzen A, Davenport RJ, Turnbaugh PJ (2001) Removing noise from pyrosequenced amplicons. BMC Bioinformatics 2011, 12:38 doi:10.1186/1471-2105-12-38 10.1186/1471-2105-12-38 PMC3045300 21276213

[CR42] Raubuch M, Beese F (1995). Pattern of microbial indicators in forest soils along an European transect. Biol Fertil Soils.

[CR43] Rousk J, Bååth E, Brookes PC, Lauber CL, Lozupone C, Caporaso JG, Knight R, Fierer N (2010). Soil bacterial and fungal communities across a pH gradient in an arable soil. ISME J.

[CR44] Schimel JP, Gulledge JM, Clein-Curley JS, Lindstrom JE, Braddock JF (1999). Moisture effects on microbial activity and community structure in decomposing birch litter in the Alaskan taiga. Soil Biol Biochem.

[CR45] Schimel J, Balser TC, Wallenstein M (2007). Microbial stress-response physiology and its implications for ecosystem function. Ecology.

[CR46] Schlichting E (1966). Blume HO.

[CR47] Schloss PD, Westcott SL, Ryabin T, Hall JR, Hartmann M, Hollister EB, Lesniewski RA, Oakley BB, Parks DH, Robinson CJ, Sahl JW, Stres B, Thallinger GG, Van Horn DJ, Weber CF (2009). Introducing Mothur: open-source, platform-independent, community-supported software for describing and comparing microbial communities. Appl Environ Microbiol.

[CR48] Sheldrick BH, Wang C, Carter MR (1993). Particle size distribution. Soil sampling and methods of analysis.

[CR49] Singh BK, Munro S, Potts JM, Millard P (2007). Influence of grass species and soil type on rhizosphere microbial community structure in grassland soils. Appl Soil Ecol.

[CR50] Tripahti BM, Kim M, Singh D, Lee-Cruz L, Lai-Hoe A, Ainuddin AN, Go R, Rahim RA, Husni MH, Chun J, Adams JM (2012). Tropical soil bacterial communities in Malaysia: pH dominates in the equatorial tropics too. Microb Ecol.

[CR51] Uhlirova E, Elhottova D, Třiska J, Šantrůčková H (2005). Physiology and microbial community structure in soil at extreme water content. Folia Microbiol.

[CR52] Wackernagel W (2006) The Various Sources and the Fate of Nucleic Acids in Soil. In: Nucleic Acids and Proteins in Soil, Nannipieri P and Smalla C (eds), Soil Biology Vol 8, Springer, pp 117 – 140

[CR53] Ward NL, Challacombe JF, Janssen PH, Henrissat B, Coutinho PM, Wu M, Xie G, Haft DH, Sait M, Badger J, Barabote RD, Bradley B, Brettin TS, Brinkac LM, Bruce D, Creasy T, Daugherty SC, Davidsen TM, DeBoy RT, Detter JC, Dodson RJ, Durkin AS, Ganapathy A, Gwinn-Giglio M, Han CS, Khouri H, Kiss H, Kothari SP, Madupu R, Nelson KE, Nelson WC, Paulsen I, Penn K, Ren Q, Rosovitz MJ, Selengut JD, Shrivastava S, Sullivan SA, Tapia R, Thompson LS, Watkins KL, Yang Q, Yu C, Zafar N, Zhou L, Kuske CR (2009). Three genomes from the phylum *Acidobacteria* provide insight into the lifestyles of these microorganisms in soils. Appl Environ Microbiol.

[CR54] Welp G (1999). Inhibitory effects of the total and water-soluble concentrations of nine different metals on the dehydrogenase activity of a loess soil. Biol Fertil Soils.

[CR55] Yeates C, Gillings MR, Davison AD, Altavilla N, Veal DA (1997). PCR amplification of crude microbial DNA extracted from soil. Letters Appl Microbiol.

[CR56] Zhang L, Xu Z (2008). Assessing bacterial diversity in soil. J Soils Sediments.

