# Supplementary material for: Soil chemical properties affect the reaction of forest soil bacteria to drought and rewetting stress
Source: Ann Microbiol. 2014 Nov 25;65(3):1627–37. doi: 10.1007/s13213-014-1002-0 (PMC4529456; doi:10.1007/s13213-014-1002-0)
Supplement: Supplementary file 1 — (DOCX 14 kb) [file 13213_2014_1002_MOESM1_ESM.docx]

Table S1 Location, texture of soils and humus forms at the sampling sites.

| Site name | Location | Texture (% ) | | | Humus form |
| --- | --- | --- | --- | --- | --- |
|  |  | 2.0 – 0.05 mm | 0.05 – 0.002 mm | < 0.002 mm |  |
| OLK1 | 50^ᵒ^17’ N, 19 ^ᵒ^29’ E | 94 | 6 | 0 | Moder |
| OLK2 | 50^ᵒ^18’ N, 19 ^ᵒ^29’ E | 94 | 6 | 0 | Moder |
| OLK3 | 50^ᵒ^19’ N, 19 ^ᵒ^32’ E | 96 | 4 | 0 | Moder |
| OLK4 | 50^ᵒ^25’ N, 19 ^ᵒ^37’ E | 96 | 4 | 0 | Moder |
| OLK5 | 50^ᵒ^32’ N, 19 ^ᵒ^38’ E | 95 | 4 | 0 | Moder |
| LEG1 | 51^ᵒ^44’ N, 16^ᵒ^04’ E | 93 | 6 | 0 | Mor-moder |
| LEG2 | 51^ᵒ^43’ N, 16 ^ᵒ^00’ E | 98 | 2 | 0 | Mor |
| LEG3 | 51^ᵒ^45’ N, 16 ^ᵒ^00’ E | 92 | 7 | 1 | Mor |
| LEG4 | 51^ᵒ^46’ N, 16 ^ᵒ^01’ E | 90 | 10 | 1 | Moder |
| LEG5 | 51^ᵒ^25’ N, 15 ^ᵒ^43’ E | 97 | 3 | 0 | Moder |
