# Supplementary material for: Soil chemical properties affect the reaction of forest soil bacteria to drought and rewetting stress
Source: Ann Microbiol. 2014 Nov 25;65(3):1627–37. doi: 10.1007/s13213-014-1002-0 (PMC4529456; doi:10.1007/s13213-014-1002-0)
Supplement: Supplementary file 2 — (DOCX 19 kb) [file 13213_2014_1002_MOESM2_ESM.docx]

Table S2. Relative shares (in %) of particular bacterial phyla in the entire bacterial population in the O horizon at different sites prior to and after the drought and rewetting stress.

| Site | Proteobacteria | | | | Acido- | Actino- | Bactero- | Chloro- | Cyano- | Firmi- | Gemmati- | Plancto- | Verruco- | Others |
| --- | --- | --- | --- | --- | --- | --- | --- | --- | --- | --- | --- | --- | --- | --- |
|  | α | β | γ | δ | bacteria | bacteria | idetes | flexi | bacteria | cutes | monadetes | mycetes | microbia |  |
|  | Prior to the stress | | | | | | | | | | | | | |
| OLK1 | 29.5 | 8.1 | 10.3 | 3.7 | 22.3 | 5.9 | 3.8 | 4.1 | 1.1 | 0.6 | 3.7 | 1.7 | 3.1 | 2.1 |
| OLK2 | 32.7 | 4.7 | 12.7 | 2.9 | 25.4 | 6.2 | 4.6 | 0.9 | 1.9 | 0.6 | 1.0 | 2.9 | 1.3 | 2.2 |
| OLK3 | 32.3 | 4.7 | 13.3 | 2.9 | 28.4 | 4.6 | 3.6 | 0.5 | 2.0 | 0.3 | 0.5 | 3.2 | 1.3 | 2.3 |
| OLK4 | 28.9 | 3.4 | 11.9 | 4.8 | 31.2 | 6.1 | 2.9 | 0.2 | 2.7 | 0.4 | 0.4 | 3.6 | 1.3 | 2.1 |
| OLK5 | 32.3 | 2.9 | 11.5 | 4.0 | 28.0 | 3.4 | 5.3 | 0.6 | 3.3 | 0.2 | 0.9 | 3.8 | 1.4 | 2.2 |
| LEG1 | 25.4 | 3.1 | 18.6 | 0.7 | 31.8 | 8.2 | 2.8 | 0.1 | 3.5 | 0.1 | 0.1 | 3.9 | 0.1 | 1.3 |
| LEG2 | 23.1 | 5.4 | 19.7 | 0.3 | 28.9 | 7.9 | 5.1 | 0.1 | 2.8 | 0.2 | 0.3 | 4.2 | 0.1 | 2.1 |
| LEG3 | 23.6 | 5.2 | 20.7 | 0.2 | 32.0 | 9.9 | 2.3 | 0.1 | 1.6 | 0.2 | 0.1 | 2.7 | 0.0 | 1.4 |
| LEG4 | 22.7 | 5.8 | 19.8 | 0.5 | 25.5 | 9.9 | 7.0 | 0.0 | 1.7 | 0.2 | 0.1 | 4.2 | 0.2 | 2.3 |
| LEG5 | 23.6 | 5.0 | 20.3 | 0.6 | 28.5 | 11.9 | 4.2 | 0.0 | 0.8 | 0.2 | 0.0 | 2.9 | 0.1 | 1.9 |
|  | After the stress | | | | | | | | | | | | | |
| OLK1 | 26.2 | 7.1 | 8.5 | 4.3 | 22.6 | 11.8 | 3.9 | 2.4 | 1.0 | 1.0 | 3.3 | 1.5 | 3.8 | 2.4 |
| OLK2 | 32.7 | 4.7 | 8.9 | 4.1 | 27.4 | 9.1 | 3.4 | 0.6 | 1.8 | 0.2 | 1.3 | 3.2 | 1.0 | 1.5 |
| OLK3 | 32.3 | 3.7 | 9.7 | 3.2 | 28.9 | 8.5 | 4.0 | 0.4 | 2.1 | 0.5 | 0.5 | 3.2 | 1.3 | 1.6 |
| OLK4 | 27.2 | 1.4 | 6.8 | 2.3 | 36.2 | 13.5 | 2.5 | 0.2 | 4.4 | 0.2 | 0.2 | 3.1 | 0.9 | 1.3 |
| OLK5 | 33.3 | 2.2 | 7.9 | 2.9 | 30.5 | 7.8 | 4.2 | 0.7 | 3.3 | 0.2 | 0.5 | 4.4 | 1.0 | 1.1 |
| LEG1 | 24.0 | 2.6 | 12.2 | 0.2 | 36.4 | 15.1 | 2.0 | 0.1 | 2.3 | 0.3 | 0.1 | 3.9 | 0.2 | 0.7 |
| LEG2 | 20.4 | 4.9 | 11.6 | 0.2 | 36.0 | 15.5 | 1.7 | 0.0 | 1.6 | 0.8 | 0.1 | 6.3 | 0.0 | 0.7 |
| LEG3 | 16.4 | 3.8 | 10.2 | 0.1 | 33.3 | 26.6 | 0.8 | 0.0 | 1.2 | 4.5 | 0.0 | 3.0 | 0.0 | 0.3 |
| LEG4 | 24.3 | 3.1 | 14.5 | 0.3 | 30.6 | 14.7 | 3.3 | 0.0 | 1.8 | 0.3 | 0.0 | 5.9 | 0.1 | 1.0 |
| LEG5 | - | - | - | - | - | - | - | - | - | - | - | - | - | - |
